# Relationship between insurance status and interhospital transfers among cancer patients in the United States

**DOI:** 10.1186/s12885-022-09242-8

**Published:** 2022-01-29

**Authors:** Muni Rubens, Venkataraghavan Ramamoorthy, Anshul Saxena, Sandeep Appunni, Subrina Sundil, Emir Veledar, Peter McGranaghan, Raees Tonse, Sergio Jose Torralbas Fitz, Michael D. Chuong, Yazmin Odia, Ritesh Kotecha, Minesh P. Mehta, Rupesh Kotecha

**Affiliations:** 1 Department of Radiation Oncology, Miami Cancer Institute, Baptist Health South Florida, Miami, Florida USA; 2 University of Central Missouri, Warrensburg, MO USA; 3 Baptist Health South Florida, Miami, Florida USA; 4 Florida International University, Miami, Florida USA; 5 Government Medical College, Calicut, Kerala India; 6 UNC Health Southeastern, Lumberton, NC USA; 7 Memorial Sloan Kettering Cancer Center, New York City, New York USA

**Keywords:** Interhospital transfer, Insurance status, Cancer hospitalization, National estimates, Affordability, Socioeconomic status, Healthcare disparity

## Abstract

**Background:**

The relationship between insurance status and interhospital transfers has not been adequately researched among cancer patients. Hence this study aimed for understanding this relationship using a nationally representative database.

**Methods:**

A retrospective analysis was conducted using National Inpatient Sample (NIS) data collected during 2010–2016 and included all cancer hospitalization between 18 and 64 years of age. Interhospital transfers were compared based on insurance status (Medicare, Medicaid, private, and uninsured). Weighted multivariable logistic regressions were used to calculate the odds of interhospital transfers based on insurance status, after adjusting for many covariates.

**Results:**

There were 3,580,908 weighted cancer hospitalizations, of which 72,353 (2.02%) had interhospital transfers. Uninsured patients had significantly higher rates of interhospital transfers, compared to those with Medicare (*P* = 0.005) and private insurance (*P* < 0.001). Privately insured patients had significantly lower rates of interhospital transfers, compared to those with Medicare (*P* < 0.001) and Medicaid (*P* < 0.001). Logistic regression analyses showed that the odds of having interhospital transfers were significantly higher among uninsured (adjusted odds ratio [aOR], 1.57, 95% CI: 1.45–1.69), Medicare (aOR, 1.38, 95% CI: 1.32–1.45) and Medicaid (aOR, 1.23, 95% CI: 1.16–1.30) patients when compared to those with private insurance coverages.

**Conclusion:**

Among cancer patients, uninsured and Medicare and Medicaid beneficiaries were more likely to experience interhospital transfers. In addition to medical reasons, factors such as affordability and socioeconomic status are influencing interhospital transfer decisions, indicating existing healthcare disparities. Further studies should focus on identifying the causal associations between factors explored in this study as well as additional unexplored factors.

**Supplementary Information:**

The online version contains supplementary material available at 10.1186/s12885-022-09242-8.

## Introduction

The United States Congress enacted the Emergency Medical Treatment and Active Labor Act (EMTALA) in 1986 [1]. The primary objective of EMTALA was to ensure that patients receive emergency medical care when needed and are not rejected or transferred between hospitals, based on affordability, insurance status, or other socioeconomic factors [1]. This law clearly defines that once patient enter the emergency departments (EDs) with any diagnosis or condition, the healthcare team should conduct a detailed examination, provide treatment, stabilize any emergency condition, and admit a patient if medically necessary. In spite of clear definitions of this law for initial evaluation, stabilization, and admission to the hospitals, if necessary (of patients seen at ER), it does not clearly provide guidance for discharge or transfer to other hospitals of patients who are already admitted to the hospital [1, 2]. In addition, interhospital transfers are often associated with adverse hospital outcomes such as increased length of stay, in-hospital mortality and hospitalization cost [3].

Despite EMTALA’s existence for more than three decades, there have been criticisms that interhospital transfers could have occurred due to patient insurance status, inconveniences for healthcare delivery team at the admitting hospital, and/or inability to recover the healthcare expenditures [4]. Though these findings have been researched primarily in EDs, there are very few studies about such occurrences in hospitalized patients. For example, in a study performed among 315,748 patients hospitalized for five common conditions, patients without medical insurances were less likely to be transferred to other hospitals, compared to patients with private medical insurances, indicating the possible existence of healthcare disparities [5]. However, similar studies, focusing specifically on hospitalized cancer patients are non-existent. Hence, the objective of our study was to examine the relationship between insurance status and interhospital transfers among hospitalized cancer patients at the national level. We hypothesized that uninsured patients are at greater risk of experiencing interhospital transfers for reasons other than optimization of treatment and management.

## Methods

### Data sources

This study is a retrospective cross-sectional analysis of data collected and stored during 2010–2016 in the National Inpatient Sample (NIS) database. The NIS is sponsored and developed by the Agency for Healthcare Research and Quality (AHRQ) as a part of the Healthcare Cost and Utilization Project (HCUP) and constitutes the largest all-payer inpatient database that records and stores discharge data [6]. Data collection methods employed by the NIS changed during our study period. Prior to 2012, the NIS collected 100% of discharge data from ~ 1000 hospitals nationwide. From 2012 onwards, the NIS started collecting stratified samples of ~ 20% of US community hospital discharge data with the objective of improving the calculation of national estimates [7]. The NIS collects data from all community hospitals such as short-term, non-Federal, and non-rehabilitation centers, while excluding Veterans Affairs and other Federal hospitals. Data collected by the NIS include several healthcare variables such as: demographics; primary and secondary diagnoses; disposition status; length of stay; hospitalization costs; and hospital characteristics.

**Fig. 1:**
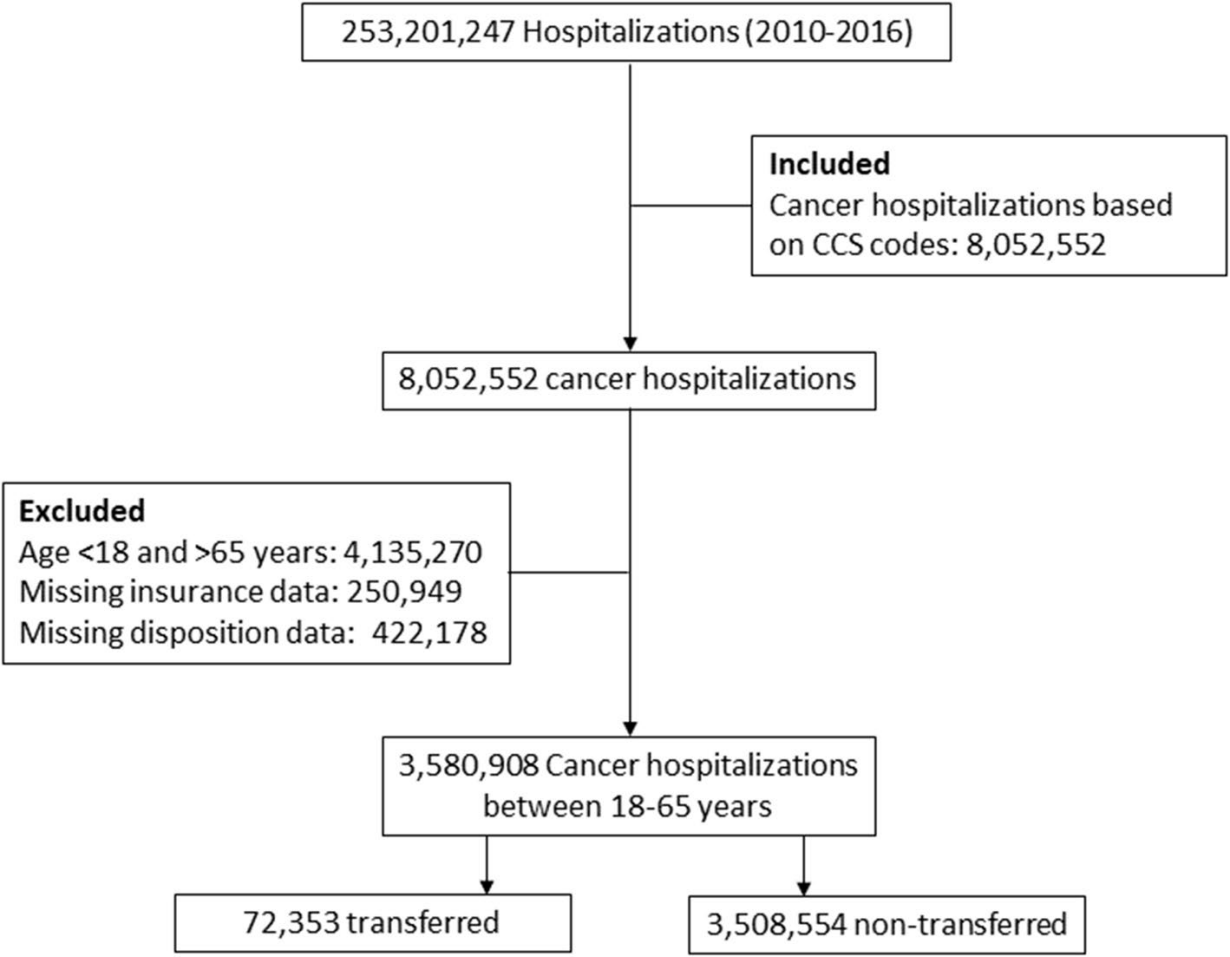
CONSORT diagram of inclusion and exclusion criteria for the study

All cancer hospitalizations aged 18–64 years were included for this analysis. Cancer hospitalizations were identified by cancer-related primary diagnosis (only initial diagnosis) Clinical Classification Software (CCS) codes 11–44 [8]. Patients ≥65 years were not included for the analysis because majority of them were insured by Medicare and their lack of variability in coverage could obscure the results of this study. Only patients who had Medicare, Medicaid, private insurance or uninsured as their primary payer were included for the analyses. Insurance coverages other than these sources were excluded because they did not constitute a homogenous group as they included several insurance coverages with very few patients. Though Medicare is primarily reserved for individuals ≥65 years of age, certain individuals with disability status such as those receiving Social Security Disability benefits for ≥24 months or those having End Stage Renal Disease or Amyotropic Lateral Sclerosis are considered eligible for Medicare coverage [9]. People under 65 years of age who are eligible for Medicaid include those with congenital disabilities as well as disabilities acquired due to illness, injury, or trauma [10]. Figure 1 shows CONSORT diagram for the study.

Patients who died during hospitalization or left the hospital against medical advice were also excluded from the analysis because they would not meet the study entry criteria (not eligible for interhospital transfer). We followed the Strengthening the Reporting of Observational Studies in Epidemiology guidelines for reporting our findings.

### Variables

The primary outcome variable in our study was discharge disposition, especially transfer to another hospital. Discharge dispositions indicating transfers to inpatient rehabilitation, home or self-care, home health care, long-term acute care, Skilled Nursing Facilities and Intermediate Care Facilities were not considered as interhospital transfers. Patient characteristics included: age; sex; race (white, black, Hispanic, and other); median household income (quartiles 1–4); and Elixhauser’s comorbidity index. Hospital characteristics included: location and teaching status (rural, urban nonteaching and urban teaching); bed size (small, medium and large); and ownership (government nonfederal, private not-profit, and private invest-own).

We followed the Strengthening the Reporting of Observational Studies in Epidemiology guidelines for reporting our findings. The study was reviewed by the Miami Cancer Institute’s Institutional Review Board, which exempted the study from institutional review board approval and waived the requirement for informed consent because it uses previously collected deidentified data stored in NIS. Informed consent was not required since this study involves an administrative database and does not contain any identifiable information that can be linked to any specific participant.

### Statistical analysis

Statistical analysis was performed using SAS (version 9.4, SAS Institute, Cary, North Carolina), which accounted for the complex survey design and clustering. As already mentioned, the NIS was redesigned in 2012 to improve national estimates. To account for this change, we used modified discharge weights for the years 2010 and 2011. Demographics, socioeconomic measures, and comorbidities were compared between patients with and without interhospital transfers using Rao-Scott chi-square test for categorical variables and independent sample t tests for continuous variables. Subsequently, we described the proportion of patients who had interhospital transfers based on hospital characteristics and insurance coverages. Weighted multivariable logistic regression analyses were used to calculate the odds of interhospital transfers based on insurance status, after adjusting for covariates such as age, sex, race, median household income, Elixhauser’s comorbidity index score, hospital location and teaching status, hospital bed size, and hospital ownership. Since the proportion of missing data was small and not missing completely on random, NOMCAR option was used during the regression analysis. Statistical significance was set at *P* < 0.05. We also conducted sensitivity analysis to identify the influence of omitted confounders on the relationship between insurance status and interhospital transfers. Details are described in the supplementary materials.

## Results

A total of 3,580,908 weighted cancer hospitalizations were selected for the analyses, of which 72,353 (2.02%) had interhospital transfers. The median age of patients significantly differed for different insurance types (Table 1). Highest proportion of females were observed in private insurance coverage, while lowest was among uninsured. Among private, Medicare and Medicaid coverages majority of the patients were Whites, followed by Blacks and Hispanics. Among uninsured patients, majority were Whites, followed by Hispanics and Blacks. The majority of the privately insured patients were in the highest income quartile, while majority of the Medicare and Medicaid coverages and uninsured were in the lowest income quartile. Highest proportion of interhospital transfers were observed among uninsured patients, followed by patients with Medicaid, Medicare, and private insurance coverages. Comorbidity levels were highest among patients with Medicare and lowest among private insurance coverages.

**Table 1:** Demographics, socioeconomic characteristics, and comorbidity status among cancer hospitalizations categorized by insurance type

Variable	Private ***n*** = 2,270,429 (63.4%)	Medicare ***n*** = 413,820 (11.5%)	Medicaid ***n*** = 680,142 (18.9%)	Uninsured ***n*** = 216,516 (6.0%)	All Insurance ***n*** = 3,580,908 (100%)	***P*** value
Age, median (IQR)	54.6 (47.5–59.6)	57.1 (51.6–61.1)	52.2 (44.1–57.9)	52.9 (45.7–58.5)	54.4 (47.1–59.5)	< 0.001
Female, % (SE)	51.4% (0.2)	49.4% (0.2)	54.1% (0.1)	48.1% (0.2)	51.5% (0.1)	
Race, % (SE)						< 0.001
White	69.6% (0.5)	61.8% (0.6)	46.4% (0.7)	49.6% (1.2)	63.1% (0.5)	
Black	9.8% (0.2)	20.1% (0.3)	21.7% (0.4)	18.0% (0.7)	13.7% (0.2)	
Hispanic	6.7% (0.2)	7.6% (0.2)	16.9% (0.7)	19.3% (0.8)	9.5% (0.2)	
Other or unknown	13.7% (0.5)	10.3% (0.5)	14.8% (0.5)	12.9% (0.7)	13.5% (0.5)	
Median household income, % (SE)						< 0.001
Quartile 1	18.8% (0.3)	37.4% (0.4)	40.0% (0.4)	37.9% (0.6)	26.0% (0.3)	
Quartile 2	22.5% (0.3)	26.4% (0.2)	26.2% (0.2)	26.7% (0.4)	23.9% (0.2)	
Quartile 3	26.9% (0.2)	21.4% (0.2)	20.9% (0.2)	21.8% (0.3)	24.8% (0.1)	
Quartile 4	31.6% (0.6)	14.5% (0.3)	12.7% (0.3)	13.5% (0.4)	25.0% (0.5)	
Interhospital transfer, % (SE)	1.7% (0.0)	2.5% (0.0)	2.6% (0.0)	2.8% (0.1)	2.0% (0.0)	< 0.001
Elixhauser’s comorbidity index, median (IQR)	1.0 (0.0–2.1)	2.2 (1.0–3.6)	1.5 (0.4–2.8)	1.2 (0.2–2.5)	1.1 (0.1–2.5)	< 0.001

*Abbreviations*: *SE* standard error, *IQR* interquartile range

Rao-Scott chi-square tests were used for comparing categorical variables and Kruskal-Wallis test for continuous variables

The median age of patients who had interhospital transfers was significantly lower than those who did not have interhospital transfers (Table 2). Female patients were significantly less likely to have interhospital transfers, compared to male patients. Black and Hispanic patients were more likely, while white patients were less likely to have interhospital transfers. Patients with median household income in the lowest quartile were more likely, while those in the highest quartile were less likely to have interhospital transfers. Elixhauser’s comorbidity index scores were significantly higher among patients who had interhospital transfers. Patients to urban teaching hospitals and large non-profit private hospitals were more likely to experience interhospital transfers.

**Table 2:** Demographics, socioeconomic characteristics, comorbidity status, and hospital characteristics among cancer hospitalizations categorized by interhospital transfer and adjusted odds of interhospital transfer based on insurance status

Variable	Interhospital transfer ***n*** = 72,353 (2.0%)	No interhospital transfer n = 3,508,554 (98.0%)	Total	***P*** value
Age, median (IQR)	53.8 (45.8–59.3)	54.4 (47.2–59.5)	54.4 (47.1–59.5)	< 0.001
Female, % (SE)	46.3% (0.4)	51.6% (0.1)	51.5% (0.1)	< 0.001
Race, % (SE)				< 0.001
White	62.2% (0.5929)	63.1% (0.6)	63.1% (0.5)	
Black	15.2% (0.4193)	13.7% (0.2)	13.7% (0.2)	
Hispanic	9.9% (0.3317)	9.5% (0.2)	9.5% (0.2)	
Other or unknown	12.5% (0.4242)	13.5% (0.5)	13.5% (0.5)	
Median household income, % (SE)				< 0.001
Quartile 1	29.4% (0.5337)	26.0% (0.3)	26.0% (0.3)	
Quartile 2	25.1% (0.4536)	23.9% (0.2)	23.9% (0.2)	
Quartile 3	23.9% (0.4123)	24.8% (0.1)	24.8% (0.1)	
Quartile 4	21.4% (0.5248)	25.1% (0.5)	25.0% (0.5)	
Elixhauser’s comorbidity index, median (IQR)	1.7 (0.5–3.0)	1.1 (0.1–2.4)	1.1 (0.1–2.5)	< 0.001
Hospital location and teaching status, % (SE)				< 0.001
Rural	14.1% (0.4)	4.1% (0.1)	4.3% (0.1)	
Urban nonteaching	38.2% (0.6)	21.8% (0.5)	22.1% (0.5)	
Urban teaching	47.5% (0.7)	73.9% (0.5)	73.4% (0.5)	
Hospital bed size, % (SE)				< 0.001
Small	19.0% (0.4)	10.8% (0.4)	11.0% (0.4)	
Medium	28.7% (0.5)	21.2% (0.5)	21.4% (0.5)	
Large	52.1% (0.7)	67.8% (0.7)	67.5% (0.7)	
Hospital ownership, % (SE)				< 0.001
Government, nonfederal	13.2% (0.6)	15.5% (0.8)	15.4% (0.8)	
Private, not-profit	73.2% (0.7)	75.2% (0.8)	75.2% (0.8)	
Private, invest-own	13.5% (0.4)	9.1% (0.3)	9.2% (0.3)	
**Association between insurance status and interhospital transfer**				
	**aOR ratio (95% CI)**			
Insurance				
Private	Reference	–	–	
Medicare	1.22 (1.16–1.29)	–	–	< 0.001
Medicaid	1.38 (1.31–1.45)	–	–	< 0.001
Uninsured	1.56 (1.45–1.69)	–	–	< 0.001

*Abbreviations: SE* standard error, *IQR* interquartile range, *aOR* adjusted odds ratio

Rao-Scott chi-square tests were used for comparing categorical variables and Kruskal-Wallis test for continuous variables

Note: The models were adjusted for age, sex, race, median household income, Elixhauser’s comorbidity index score, hospital location and teaching status, hospital bed size, and hospital ownership. Complete model results are available from the authors upon request

Logistic regression showed that the odds of having interhospital transfers were significantly higher among uninsured (adjusted odds ratio [aOR], 1.57, 95% CI: 1.45–1.69), Medicare (aOR, 1.38, 95% CI: 1.32–1.45) and Medicaid (aOR, 1.23, 95% CI: 1.16–1.30) patients when compared to those with private insurance coverages (Table 2).

Table 3 shows the comparison of interhospital transfer rates for different coverages. Uninsured patients had significantly higher rates of interhospital transfers when compared to patients with Medicare (*P* = 0.005) and private insurance (*P* < 0.001). Privately insured patients had significantly lower rates of interhospital transfers when compared to patients with Medicare (*P* < 0.001) and Medicaid (*P* < 0.001).

**Table 3:** Comparison of interhospital transfer rates among different insurance types

Insurance status	Proportion of interhospital transfer	***P*** value
Uninsured	2.8%	
versus Private	1.7%	< 0.001
versus Medicare	2.5%	0.005
versus Medicaid	2.6%	0.075
Private	1.7%	
versus Medicare	2.5%	< 0.001
versus Medicaid	2.6%	< 0.001

Rao-Scott chi-square tests were used for comparing categorical variables

## Discussion

Since the 1980s, congressional acts have been put into place to provide key protections to vulnerable patients. This includes the ability to receive adequate hospital care as well as making sure that interhospital transfer decisions are not based on affordability, insurance status, or other socioeconomic factors. As cancer patients are starting to make up a significant proportion of hospital admissions and healthcare expenditures, the purpose of this study was to examine interhospital transfers for this subgroup of patients. Therefore, in this study we utilized the NIS, the largest all-payer inpatient database in the United States, to find whether interhospital transfers among cancer patients were significantly influenced by insurance status, after adjusting for covariates such as demographics, comorbidities, and hospital characteristics. Our results show that these transfers were significantly higher among the most vulnerable of patients who were uninsured, and Medicare and Medicaid patients, compared to those with private coverages.

The findings in our study should be viewed in the context of ambiguities inherent in the EMTALA, enacted in 1986. EMTALA mandates that EDs in hospitals should provide emergency investigations and treatments for stabilizing patients and admit them to hospitals, if necessary, irrespective of their financial status and affordability [11, 12]. However, the mandates of EMTALA do not apply for hospitalized patients who are already admitted or for interhospital transfer decisions. There is also uncertainty about how much care should be provided by the hospitals once medical stabilization has been achieved. These ambiguities could be responsible for basing affordability factors such as insurance coverages and socioeconomic factors while making decisions on interhospital transfers. The findings in our study suggest that regulations for preventing affordability and socioeconomic factors from influencing interhospital transfer decisions could significantly decrease such incidences.

The findings in our study parallel the trends reported in prior studies performed in the ED setting. For example, in a study that analyzed transfer requests to a tertiary care center from a community hospital ED, there were higher efforts for transferring uninsured patients [13]. Similarly, a cross-sectional study that analyzed 215,028 ED visits for respiratory diseases such as pneumonia, chronic obstructive pulmonary disease, and asthma, uninsured patients and Medicaid beneficiaries were significantly more likely to have interhospital transfers [14]. In a study by Rosenbaum et al. that evaluated the enforcement of EMTALA act through a compilation of case studies, it was found that community hospital EDs were increasingly transferring uninsured patients to public hospital without adequate evaluations and treatments [15]. A study done by Delgado et al. among patients with major trauma and arriving at non-trauma EDs showed that insured patients were less likely to be transferred to tertiary care centers and hence at increased risk of receiving suboptimal trauma care [16]. These findings suggest that financial factors such as insurance coverage and affordability could be determining factors for interhospital transfers. Though these studies showed findings similar to ours, it should be noted that they reflected referrals from EDs and not interhospital transfers like our study. Only one study published recently by Hanmer et al. looked for interhospital transfers among hospitalized patients [5]. This study reported that, for five common medical conditions such as biliary tract disease, chest pain, pneumonia, septicemia, and skin infections, uninsured patients were less likely to be transferred to other hospitals, compared to patients with private coverages. This finding is contrary to the finding in our study where interhospital transfers were higher among uninsured patients probably due to higher cost associated with cancer hospitalizations. Nevertheless, the study by Hanmer et al. hypothesizes that there could be alternate factors responsible for this unusual finding. Except for extreme emergencies, receiving hospitals could have increasingly scrutinized insurance status before accepting patients from transferring hospitals [5]. There could be complex dynamics functioning between transferring and receiving hospitals, wherein receiving hospitals could have strongly resisted accepting uninsured patients, resulting in lower interhospital transfers among uninsured patients [5]. Other factors such as patient complexities and patient choices could have also influenced interhospital transfers and may be responsible for the unusual finding in this study [5]. Thus, a number of factors could be affecting the relationship between insurance status and interhospital transfers and future studies should focus on understanding them.

It could also be possible that in our study, cancer patients with higher disease severity could have experienced greater levels of interhospital transfer for advanced and varying treatment options. However, we were not able to qualify cancer severity due to unavailability of data about grading and staging of cancers. Finally, there could be a number of confounding variables which could not be included in the regression model due to limitations in the availability of the variables. However, we conducted a sensitivity analysis to identify the effect of unmeasured confounders on the relationship between insurance status and interhospital transfers.

Though our analysis showed that uninsured, Medicare and Medicaid patients were more likely to be transferred, there are other factors that should be considered such as the directionality of the transfers. For example, when transfers are recommended from institutions with greater levels of care, uninsured patients could be at risk of not receiving optimum levels of care. On the contrary, when transfers are recommended from institutions with lower levels of care, uninsured patients could be at risk of excessive financial spending due to sophisticated procedures and treatments with few tangible benefits, when in fact they cannot even afford existing levels of care provided by the referring institutions. Both these situations indicate the existence of healthcare disparities, wherein, socioeconomic factors such as having better insurance coverages improve the chances of receiving optimum levels of care. In spite of these assumptions, understanding the relationship between insurance status, interhospital transfers and expensive procedures and treatments is difficult because transferred patients have higher levels of quantifiable and non-quantifiable case complexities, when compared to non-transferred patients [17].

In spite of these findings, our study has some limitations. The NIS constitutes an administrative database and does not collect information about the choices made by the patients which could have directly or indirectly influenced interhospital transfers. Therefore, the role of patients’ own decisions for these transfers could not be determined. Similarly, the NIS does not collect detailed clinical information such as grading and staging of cancers and hence the influence of case severity and appropriateness of interhospital transfers could not be determined. Severity of cancer in terms of grading and staging are important clinical factors that could have profound implications on clinical decisions affecting interhospital transfers. The database does not contain information about pre-hospital triage and observation or the transferred hospital. The database also deletes all patient identifiers for confidentiality. These missing information makes it impossible to connect patient data across different settings such as EDs, observation wards, and receiving and transferring hospitals, thereby limiting our understanding of the interplay of factors responsible for interhospital transfers. In addition, we could not ascertain whether interhospital transfers were recommended for sustaining the continuity of care due to lack of data. Finally, NIS database deleted all personal identifiers to ensure confidentiality of the collected data. Patients who were readmitted were considered as independent new admissions, thus obliterating the differences between index cases and readmitted cases. This could have overestimated hospitalization rates in our study.

## Conclusion

Our study showed that among cancer patients, uninsured and those with Medicare and Medicaid were more likely to be transferred from one hospital to another. This shows that factors other than medical reasons such as affordability and socioeconomic status could be influencing interhospital transfer decisions, indicating existing healthcare disparities. Interhospital transfers among cancer patients should be investigated further to identify the causal associations between factors explored in this study as well as additional unexplored factors.

## Supplementary Information

**Additional file 1: Figure S1**. Sensitivity analysis results for the effect, ATE, of insurance on transfer. **Figure S2**. Sensitivity analysis results for the effect, ATT, of insurance on transfer.

## Data Availability

The dataset used in this study is publicly available for purchase from https://www.hcup-us.ahrq.gov/nisoverview.jsp
