# Supplementary material for: Relationship between insurance status and interhospital transfers among cancer patients in the United States
Source: BMC Cancer. 2022 Jan 29;22:121. doi: 10.1186/s12885-022-09242-8 (PMC8801067; doi:10.1186/s12885-022-09242-8)
Supplement: Supplementary file 1 — Additional file 1: Figure S1. Sensitivity analysis results for the effect, ATE, of insurance on transfer. Figure S2. Sensitivity analysis results for the effect, ATT, of insurance on transfer. [file 12885_2022_9242_MOESM1_ESM.docx]

**Supplementary Information**

**Relationship between Insurance Status and Interhospital Transfers among Cancer Patients in the United States**

Muni Rubens PhD, Venkataraghavan Ramamoorthy PhD, Anshul Saxena PhD, Subrina Sundil MD, Emir Veledar PhD, Peter McGranaghan MS, Sergio Jose Torralbas Fitz MD, Ana Viamonte-Ros MD, Yazmin Odia MD, Ritesh Kotecha MD, Minesh P Mehta MD, Rupesh Kotecha MD

**Sensitivity Analyses for Unmeasured Confounders**

**Causal Inference Framework**

Outcome variable is denoted by Y (variable “transfer” [yes or no] in our data), treatment assignment by Z (variable “insurance” [private, Medicare, Medicaid, and uninsured] in our data), and a set of covariates by X (age, gender, race, year, Elixhauser’s comorbidity index, median household income, hospital ownership, hospital bed size, and hospital location and teaching status). In the case where all confounders are measured, we could be confident to assume ignorability, i.e. independence between the potential outcomes and the treatment variable, conditional on a set of covariates in the causal inference framework:

$$Y Ʇ Z | X$$

However, the assumption that all confounders are measured may fail. As a strategy for exploring the sensitivity of this assumption, we posit an unmeasured variable, U, which if included, will satisfy the ignorability assumption:

$$Y Ʇ Z | X, U$$

With this assumption, we can have

$$E\left[ Y\left( Z \right) | X,U \right]=\beta^{y}X+\xi^{y}U+\tau Z$$

Where $\beta^{y}$ is the vector of regression coefficients of X, $\xi^{y}$ the coefficient of U, and $\tau$ the treatment effect. Our goal is to understand the treatment effect estimate we would have obtained if we conditioned on a posited unmeasured confounder U. We applied the method proposed by Dorie et al.^1^ to assess sensitivity to unmeasured confounding.

**Algorithm**

The strategy for recovering treatment effects conditional on U requires generating a potential confounder $\tilde{U}$ consistent with the stated complete data likelihood so we could estimate the true treatment effect by conditioning on both X and the generated $\tilde{U}$. In the causal inference framework, we have:

$$Y|X,U,Z \sim N\left( \beta^{y}X+\xi^{y}U+\tau Z, \sigma^{2} \right) (1)$$

$$Z|X,U \sim Bernoulli(\phi\left( \beta^{z}X+\xi^{z}U \right)) (2)$$

$$U \sim Bernoulli(\pi)$$

for the case of binary treatment.

The distribution of U conditional on the sensitivity parameters and observed data corresponding to this complete data likelihood is not as readily available as in the multivariate normal case. Thus, we relied on a computational method to draw from the correct distribution. This strategy capitalizes on the fact that if the parameters from the complete data likelihood were known, the conditional distribution of U would be straightforward to define as a Bernoulli distribution with probabilities equal to the ratio of appropriate likelihoods:

$$U|Y,Z,X \sim Bernoulli\left( \frac{\pi^{y,z,x,u=1}}{\pi^{y,z,x}} \right) (3)$$

To this situation, Bayesian method can be applied.^1^ In summary, for any valid combination of sensitivity parameters, $\xi^{z}$ and $\xi^{y}$, we would obtain an estimate of the treatment effect, $\tau$, through sampler from posterior sampler.

Two conditional average treatment effects that are often of interest are the average treatment effect on the treated (ATT) and the average treatment effect on the controls (ATC), given by $E[Y(1)-Y(0) \mid Z = 1]$ and $E[Y(1) - Y(0) \mid Z = 0]$, respectively. Note that for the ATT (ATC), the difference in potential outcomes is averaged only over those units observed to be in the treatment (control) group. Average treatment effect (ATE) can be found by averaging the conditional expectation E[Y(1) - Y(0) ∣ X] =E[Y(1) ∣ Z = 1, X] - E[Y(0) ∣ Z = 0, X] over the distribution of X.

**Sensitivity Analysis on Effect of Insurance on Transfer**

Figure S1 is the contour plot based on the sensitivity analysis of ATE. The primary result is given by the black contours. Each of these represents the combinations of sensitivity parameters for U that lead to the same estimated treatment effect (that is, the estimated coefficient on Z in the regression of Y on Z, X, and U, noted along the contour). For instance, sensitivity parameter pairs ($\xi^{z}=$ -0.5, $\xi^{y}=$ 0.3) and ($\xi^{z}=$ -0.1, $\xi^{y}=$ 0.6) both (approximately) fall on a contour corresponding to a treatment effect estimate of 0.21. The red curve represents the contour along which the treatment effect estimate is reduced to zero. The thin black lines report the basic effect, while the colored lines highlight specific levels of interest. Specifically, the blue lines labeled with ‘N.S.’ demarcate the point at which the estimated effect is no longer statistically significant at the 5% level. Finally, the thick gray line corresponds to the treatment effect estimate that would arise with an unmeasured confounder whose strength is equivalent to that of the covariates whose marginal effect sizes are of the greatest magnitude. The naïve treatment effect estimate is reported next to the horizontal line at the base of the y axis, in the lower right of the plot.

The plot suggests that absent unmeasured confounding effect estimate would be about -0.15 (odd ratio = exp(-0.15) = 0.86, meaning that privately insured patients were 0.86 times less likely to transfer). It further suggests that it would take a weak unmeasured confounder that was negatively associated with treatment but positively associated with the outcome to yield a significant positive effect (such effects correspond to the upper-left corner of the plot). On the other hand, if the unmeasured confounder were reasonably strongly and positively associated with both treatment and outcome, this would suggest a significant negative treatment effect estimate (upper-right corner of the plot).

The blue N.S. line indicates that either fairly strong positive association between the unmeasured confounder and the response or fairly strong negative association between the unmeasured confounder and the treatment could lead to non-significant treatment effect.


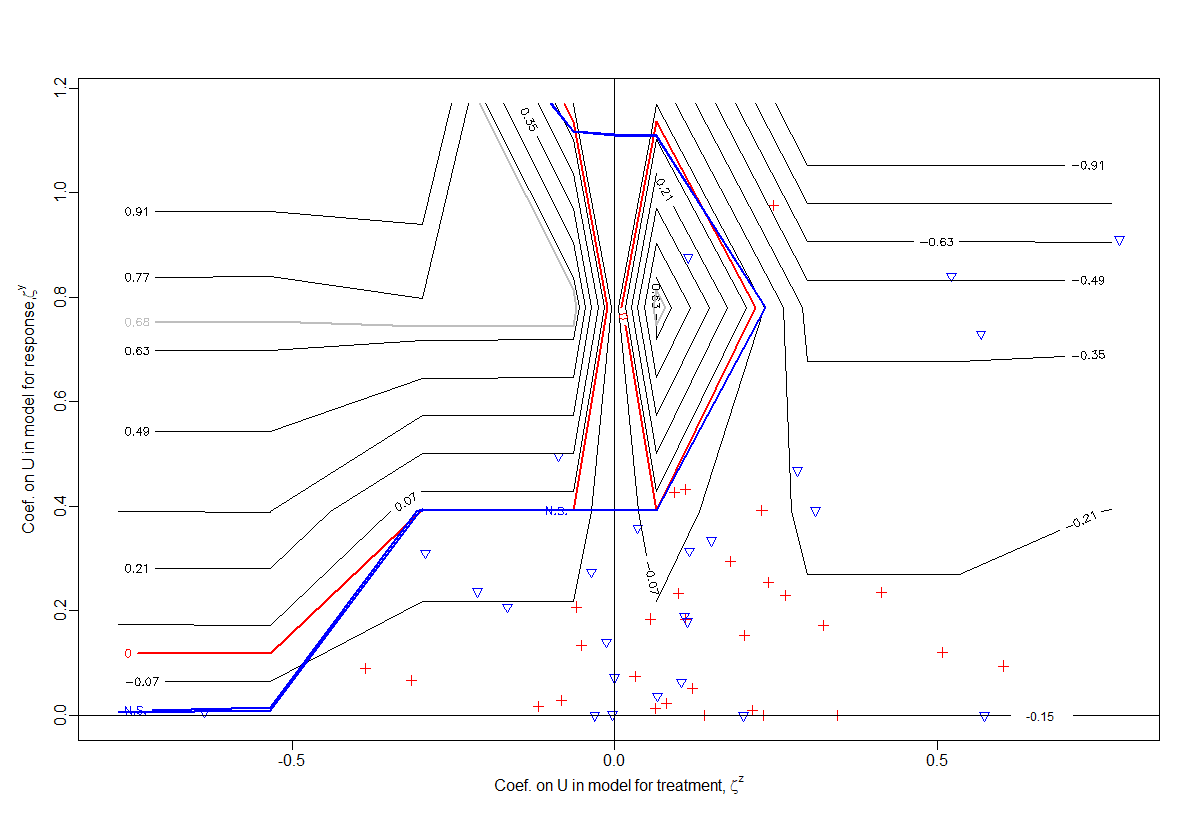


Figure S1. Sensitivity analysis results for the effect, ATE, of insurance on transfer


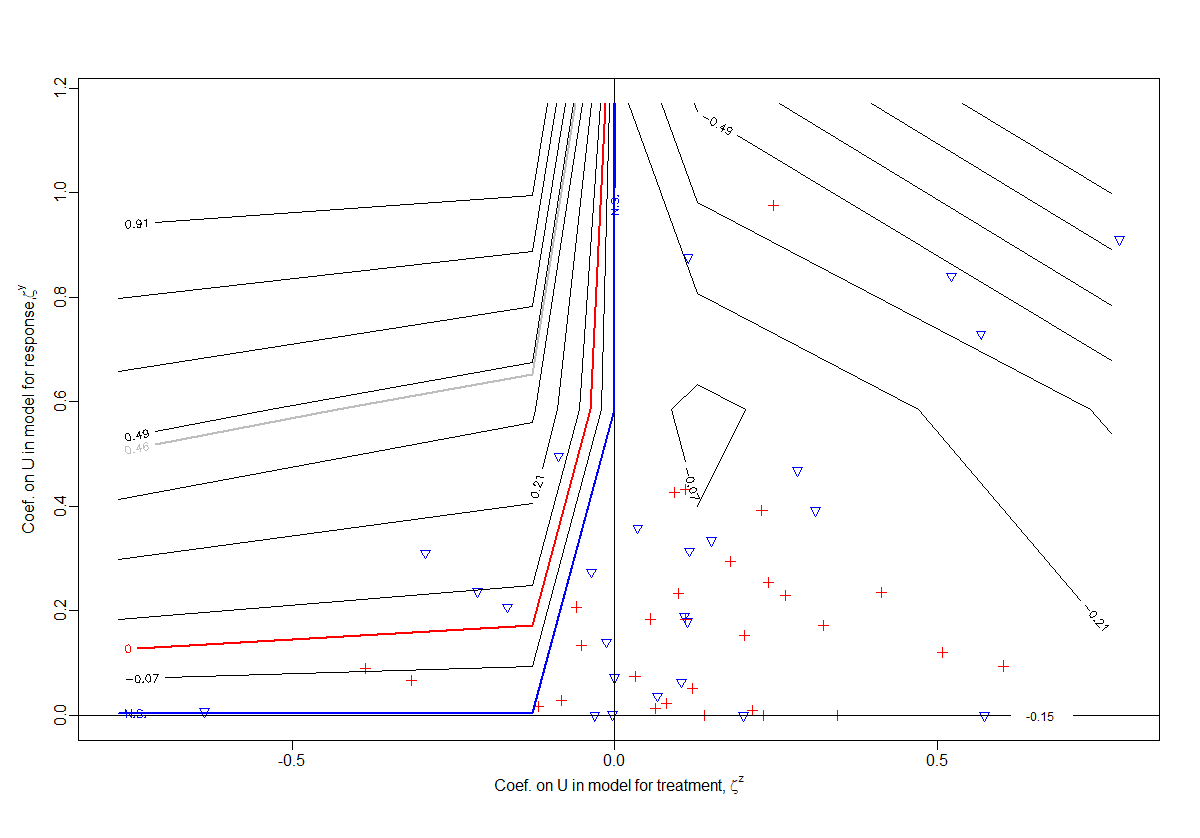


Figure S2. Sensitivity analysis results for the effect, ATT, of insurance on transfer.

**Reference**

1. Dorie V, Harada M, Carnegie NB, Hill J. A flexible, interpretable framework for assessing sensitivity to unmeasured confounding. Stat Med. 2016 Sep 10;35(20):3453-70.
